# Automatic classification of pediatric pneumonia based on lung ultrasound pattern recognition

**DOI:** 10.1371/journal.pone.0206410

**Published:** 2018-12-05

**Authors:** Malena Correa, Mirko Zimic, Franklin Barrientos, Ronald Barrientos, Avid Román-Gonzalez, Mónica J. Pajuelo, Cynthia Anticona, Holger Mayta, Alicia Alva, Leonardo Solis-Vasquez, Dante Anibal Figueroa, Miguel A. Chavez, Roberto Lavarello, Benjamín Castañeda, Valerie A. Paz-Soldán, William Checkley, Robert H. Gilman, Richard Oberhelman

**Affiliations:** 1 Department of Global Community Health and Behavioral Sciences, Tulane University School of Public Health and Tropical Medicine, New Orleans, Louisiana, United States of America; 2 Bioinformatics and Molecular Biology Laboratory, Department of Cellular and Molecular Sciences, Faculty of Science, Universidad Peruana Cayetano Heredia, Lima, Peru; 3 Research and Development Laboratory, Science and Philosophy Faculty, Universidad Peruana Cayetano Heredia, Lima, Perú; 4 Infectious Diseases Research Laboratory, Department of Cellular and Molecular Sciences, Universidad Peruana Cayetano Heredia, Lima, Peru; 5 Unidad De Rehidratación Oral. Instituto Nacional de Salud del Niño, Lima, Peru; 6 Biomedical Research Unit, Asociación Benéfica Prisma, Lima, Peru; 7 Laboratorio de Imágenes Médicas, Sección Electricidad y Electrónica, Departamento de Ingeniería Pontificia Universidad Católica del Perú, San Miguel, Lima, Perú; 8 Division of Pulmonary and Critical Care, School of Medicine, Johns Hopkins University, Maryland, United States of America; 9 Program in Global Disease Epidemiology and Control, Bloombeg School of Public Health, Johns Hopkins University, Maryland, United States of America; 10 Department of International Health, Johns Hopkins Bloomberg School of Public Health, Baltimore, Maryland, United States of America; Public Library of Science, UNITED KINGDOM

## Abstract

Pneumonia is one of the major causes of child mortality, yet with a timely diagnosis, it is usually curable with antibiotic therapy. In many developing regions, diagnosing pneumonia remains a challenge, due to shortages of medical resources. Lung ultrasound has proved to be a useful tool to detect lung consolidation as evidence of pneumonia. However, diagnosis of pneumonia by ultrasound has limitations: it is operator-dependent, and it needs to be carried out and interpreted by trained personnel. Pattern recognition and image analysis is a potential tool to enable automatic diagnosis of pneumonia consolidation without requiring an expert analyst. This paper presents a method for automatic classification of pneumonia using ultrasound imaging of the lungs and pattern recognition. The approach presented here is based on the analysis of brightness distribution patterns present in rectangular segments (here called “characteristic vectors“) from the ultrasound digital images. In a first step we identified and eliminated the skin and subcutaneous tissue (fat and muscle) in lung ultrasound frames, and the “characteristic vectors”were analyzed using standard neural networks using artificial intelligence methods. We analyzed 60 lung ultrasound frames corresponding to 21 children under age 5 years (15 children with confirmed pneumonia by clinical examination and X-rays, and 6 children with no pulmonary disease) from a hospital based population in Lima, Peru. Lung ultrasound images were obtained using an Ultrasonix ultrasound device. A total of 1450 positive (pneumonia) and 1605 negative (normal lung) vectors were analyzed with standard neural networks, and used to create an algorithm to differentiate lung infiltrates from healthy lung. A neural network was trained using the algorithm and it was able to correctly identify pneumonia infiltrates, with 90.9% sensitivity and 100% specificity. This approach may be used to develop operator-independent computer algorithms for pneumonia diagnosis using ultrasound in young children.

## Introduction

Pneumonia is one of the commonest causes of death among children under five years of age[1], with its highest case-fatality rate among infants in the post-neonatal period. Worldwide, it disproportionately affects the poorest populations in underserved regions[1], where important barriers in access to rapid diagnostics, proper care and treatment are often lacking.

Pneumonia, in the absence of complicating medical conditions or viral etiologies, responds to simple and effective treatment with antibiotics, with good outcomes in most cases receiving prompt and appropriate treatment [2–4]. However, deaths from pneumonia still happen due to delays in diagnosing both the condition and its severity [5]. In 1984 the World Health Organization (WHO) introduced the standardized case-management for pneumonia, based on age-specific respiratory rates and integrated in a program for the control of acute respiratory infections [6]. These WHO criteria were initially meant to be used by community workers [7] but due to challenges in counting respiratory rates, misclassification is common and frequently there is an overlap with other respiratory diseases such as asthma or bronchiolitis that do not benefit from antibiotics. Moreover, in spite of subsequent targeted initiatives, such as the Global Action Plan for the Prevention and Control of Pneumonia, a framework developed by the WHO and the United Nations Children’s Fund [8], the reduction in pneumonia-related mortality was not as much as anticipated (two thirds between 2000 and 2015), and deaths attributable to pneumonia still represent 1 in 5 of total deaths in children under age 5 [1].

**Fig 1:**
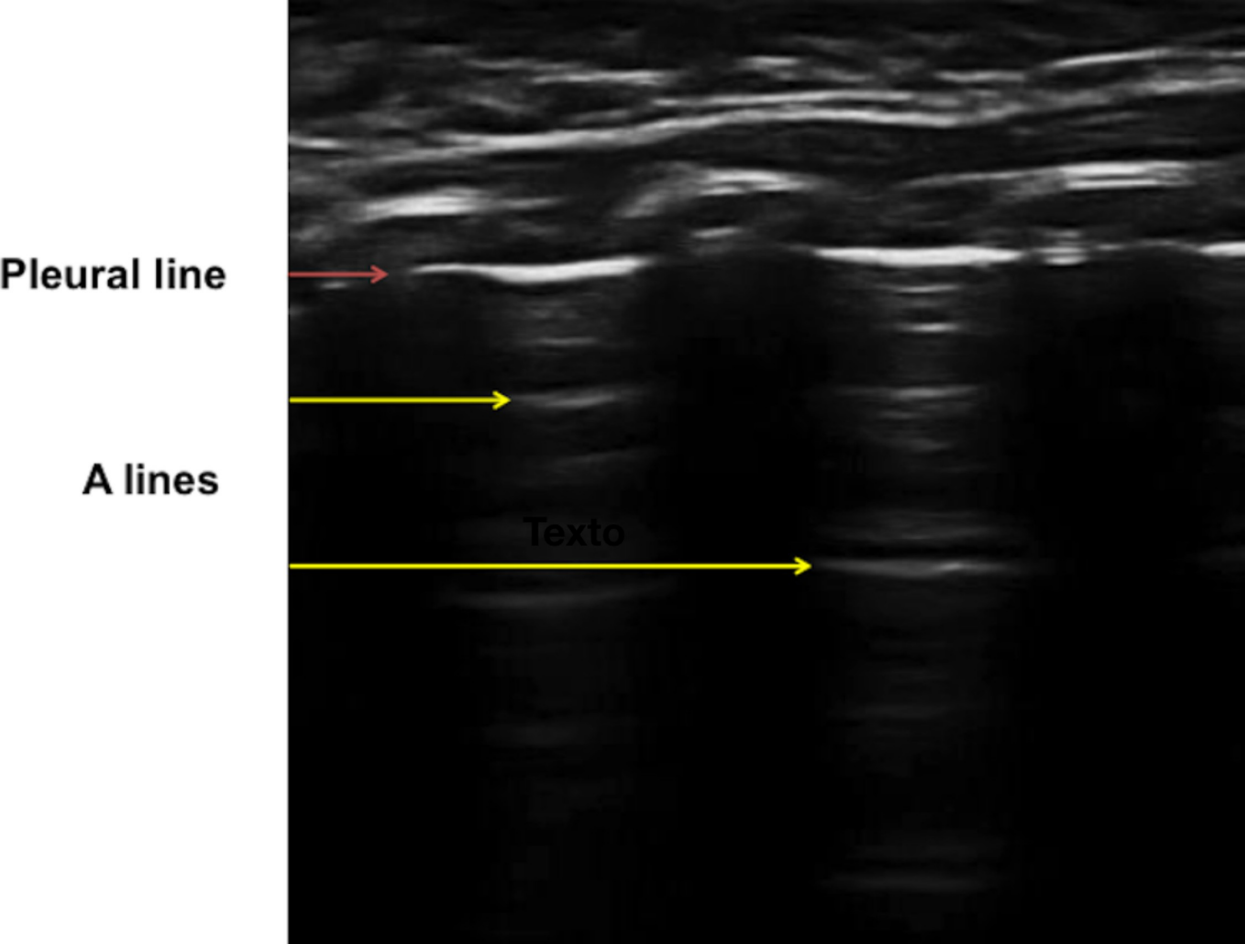
Ultrasound of the lung with the echogenic pleural line and the horizontal artifacts “A lines“.

Thoracic ultrasound is becoming a useful and readily available technique for physicians assessing a variety of respiratory, hemodynamic and traumatic conditions [9–11]. While air in the lungs makes ultrasound examination more challenging than it is for solid organs, lung patterns that allow detection of pulmonary pathology arise from the pleura (or pleural line) [12], which delineates the lung surface or border. In the normal lung, there is a great difference in impedance between the pleura and the alveolar air, which causes ultrasound waves to be completely reflected [13]. Nonetheless, ultrasound waves retain high-energy concentrations and are thus reflected several times between the acoustic impedance surface and the ultrasound transducer; this produces multiple transverse echoes that are displayed on the monitor at equidistant intervals and increasingly greater depths with decreasing intensity. These horizontal repetitions of the pleural line at regular intervals, produce a typical pattern, the so-called “A lines”[11, 14] (Fig 1).

On the other hand, when the subpleural alveoli contain fluid, semifluid or solid material (inflammatory exudate), the ultrasound waves pass through the pleura and reach the area of parenchymal consolidation; so the echostructure of parenchymal consolidation is often heterogeneous (lacking the regular A line pattern), with coexisting areas of parenchymal compactness and echogenic reflection [15]. Pneumonia, therefore, is a water-rich pulmonary consolidation that allows for good ultrasound transmission. Furthermore, in adults the infiltrate abuts the pleura in 98.5% of cases, making it easily detected by pulmonary ultrasound; in children, that is likely to happen at least as often as in adults, due to a smaller pulmonary volume [16]. Ultrasound will detect the vast majority of infiltrates, with a sensitivity of 90% and specificity of 98.5% in adults [15].

While the aforementioned features and advantages make ultrasound an excellent alternative to conventional imaging diagnosis in hospital settings, it still requires specialized equipment and trained healthcare personnel to perform and interpret the results, and none of these are commonly available in low-resource settings. Therefore, in order to facilitate the diagnostics of pneumonia in low-resource settings, it would be useful to have an automatic system to assist the interpretation of ultrasound images.

Artificial intelligence based on artificial neural networks is a common approach for automatic computer learning and classification [17]. Based on the measurable characteristics of a particular phenomenon after a training and validation step based on a selected dataset, it can assign a classification that can be used as the basic of a diagnosis [18]. In this study we present an original method of pneumonia recognition based on a computerized algorithm that allows for automatic recognition of pulmonary infiltrates. This system could help non-expert technicians, such as health auxiliaries or community workers to detect pneumonia in low-resource settings, where specialized personnel to interpret ultrasound images is not available.

## Materials and methods

### Study population

Children under 5 years of age admitted with a diagnosis of lobar pneumonia made by a pediatrician after clinical examination and chest X-ray (n = 15) and well children without any pulmonary comorbidity or cardiac condition (n = 6) were invited to participate in this study. Those whose parents or caregivers agreed to participate were enrolled and evaluated by pulmonary ultrasound during the first days of stay (pneumonia cases) and as outpatients for well children. In all cases, signed informed consent was obtained from the child ‘s parents or legal guardian. The study protocol and informed consents were approved by institutional review boards at Instituto Nacional de Salud del Niño (INSN), and the Johns Hopkins Bloomberg School of Public Health.

### Data collection: Ultrasound exam procedures

Data were collected by a trained physician using an Ultrasonix SonixTouch device with a linear probe L14-5/38 (Vancouver, British Columbia, Canada) following an ad-hoc procedure, as follows. The evaluation protocol consisted of dividing the thorax in 12 regions: 4 frontal, 4 posterior and 4 lateral. For frontal regions, the transducer was placed along a mid-clavicular line, and the probe was always placed 90 degrees from the skin and from the pleura. A transversal line on the 5th intercostal space divided the anterior thorax in 2 halves: superior and inferior; 2 left and 2 right anterior quadrants were evaluated (one superior and one inferior quadrant on each side). For the 4 posterior regions, each hemithorax was divided again in superior and inferior halves according to a transversal line coinciding with the inferior angle of the shoulder blade creating 2 left and 2 right posterior regions (superior and inferior). Finally, the lateral regions were evaluated along the medial line that goes from the upper middle axilla up to the diaphragm, and was divided into superior or inferior segments depending on whether it was located above or below the 5th intercostal space.

### Viewer classification

**Fig 2:**
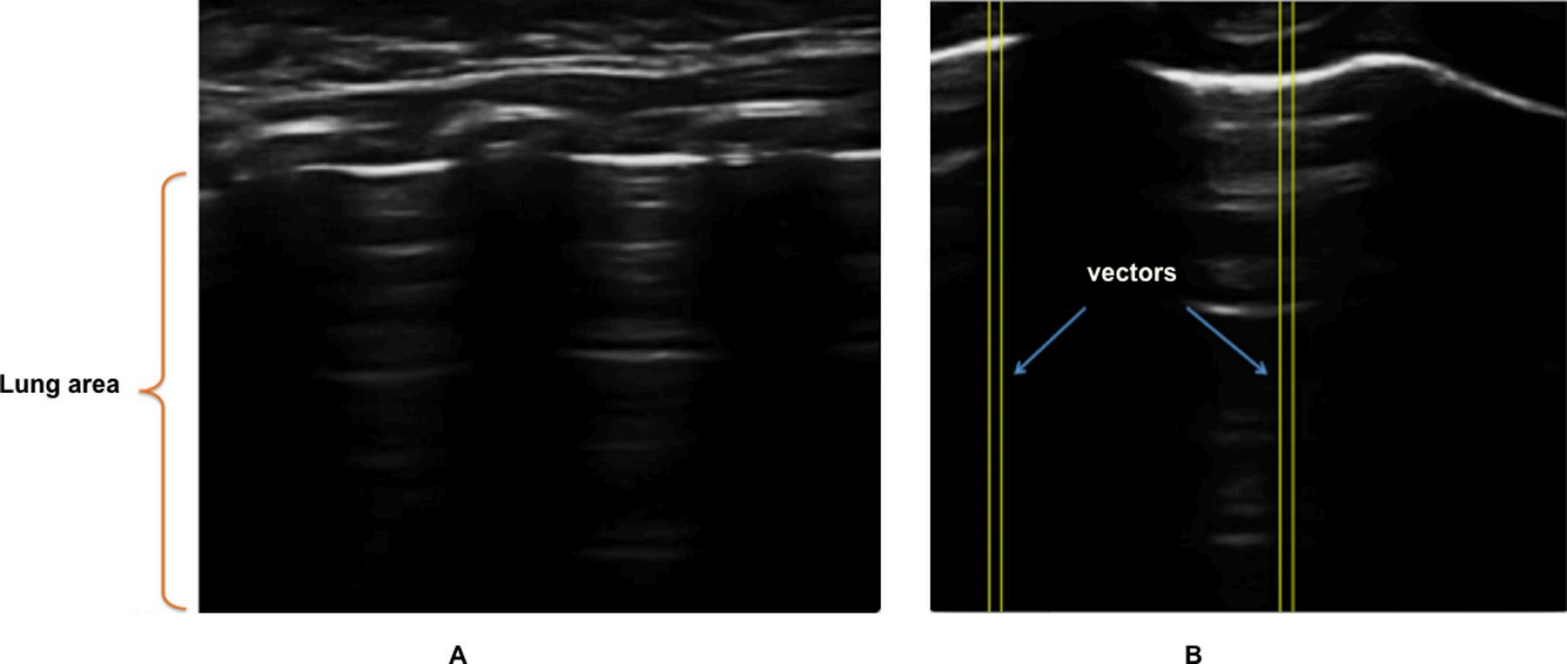
(A) Ultrasound of the lung, (B) Segmentation of (A) in vectors.

Lung ultrasound data (example shown in Fig 2A) from the recorded videos were visually previewed and the viewer–an expert trained in the international guidelines- made a classification as positive or negative according to their evidence of pneumonia infiltrate as follows: each ultrasound frame (of 8-bits, 256 levels of gray scale) was subdivided into 400 linear rectangular sections, of 10 pixels width each. These rectangular sections extend from the surface of the skin to the bottom of the frame, and are called “vectors” hereafter (Fig 2B). From these two groups of frames (positive and negative for pneumonia), vectors were manually classified into three categories (pneumonia, healthy lung, and rib bone), depending if they corresponded to a region of a pneumonia infiltrate, a normal lung, or to the acoustic shadow of the rib respectively. Complete blockage of acoustic signals by rib bones result in linear segments of opacification on lung ultrasound, starting from the top of the rib bone and continuing to the base of the image. Lung patterns indicating pathology are discernable in the intercostal spaces, between these segments of opacification. The selection of 10 pixels width responded to a preliminary analysis, where the pattern of average brightness across the 10 pixels was plotted along the complete depth of the image. Two independent observers agreed that these patterns averaged across 10 pixels width were informative enough.

### Imaging processing

#### Pleural line identification and skin/soft tissue removal

**Fig 3:**
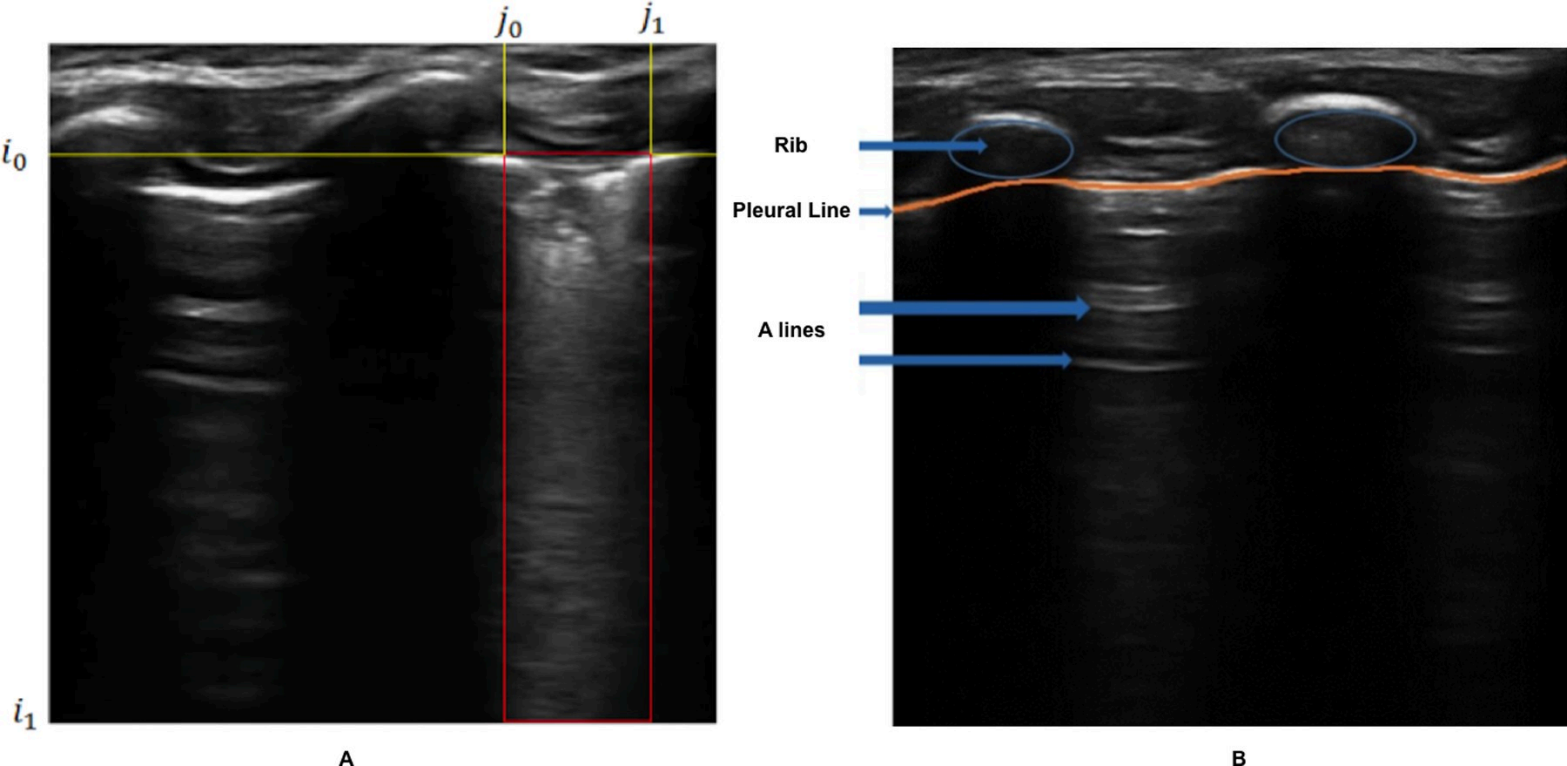
(A) Selection of the analysis zone. (B) Application of the skin filter (segmentation of the area above the pleural line).

The areas of interest for identifying pneumonia consolidation are located between two consecutive acoustic shadows of the ribs, below the pleural line in the intercostal regions (Fig 3A), therefore the contribution to the classification of pneumonia of the area above the pleural line, corresponding to the skin and soft tissues, is negligible.

In order to simplify the analysis, we recognized and discarded the skin and soft tissues portion of the frames. For this we applied a filter to remove all noise caused by the extra pulmonary soft tissues, associated with a concentration of high-brightness pixels located above the pleura line (Fig 3B). The details of this procedure are available at: https://osf.io/hmr3w/.

To evaluate the performance of the pleural line identification and skin-removal procedure by our computerized algorithm, a total of 60 frames (30 with evidence of pneumonia consolidate and 30 with no evidence of pneumonia consolidate), were classified by a human expert analyst, and simultaneously processed with the algorithm to remove the skin portion. For all the 60 frames, the expert analyst manually recognized the pleura line and removed the skin portion, using image-processing software Image J [19]. The pleural line obtained by the algorithm was compared with the pleural line manually, recognized by the expert analyst, and the mean quadratic error from both traces was calculated. For all further steps detailed below, the original frames with the skin removed, were used.

#### Extraction of features: Brightness profiles

**Fig 4:**
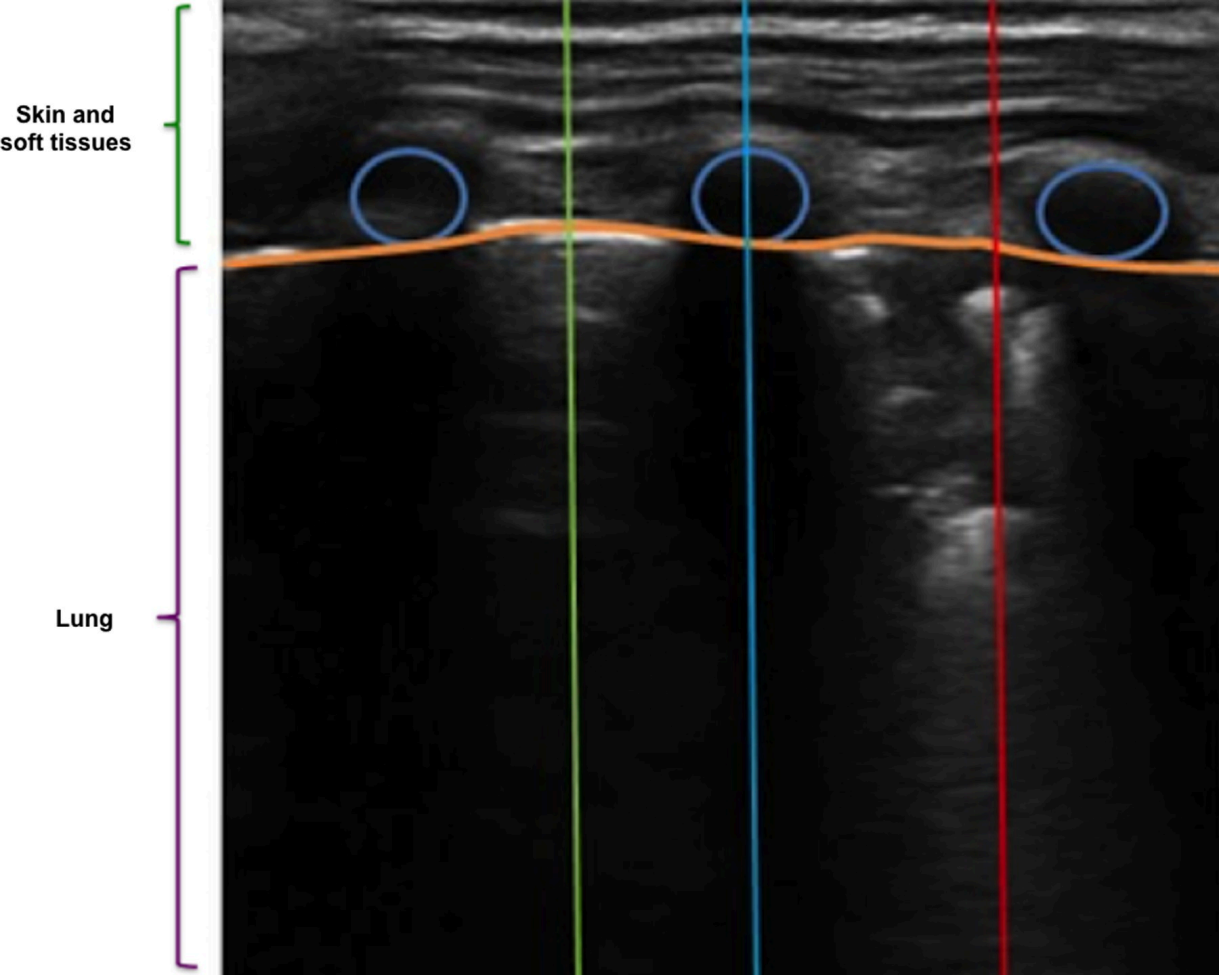
Examples of vectors in specific regions used to compute brightness profiles: healthy (green), rib-bone (blue), and pneumonia (red).

The pattern of the brightness profile of vectors was the principal source of information to identify pneumonia consolidates. The brightness profile of each vector was determined by the distribution of the averaged brightness across the 10-pixels width of the vector at each position along its length (vertical column perpendicular to the skin surface; Fig 4).

The features that we considered in this analysis were the average brightness of the sections of the vectors corresponding to the 25, 50, 75 and 100 percentile of the length, starting from the first element near the pleural line towards the last one (Features 1 or F1) or vice versa, starting from the last element and working up to the pleura (Features 2 or F2; more details on F1 and F2 calculations section available at: https://osf.io/hmr3w/).

### Training and evaluation of an artificial neural network algorithm

An artificial neural network is a computer learning system that mimics a biological central nervous system. Mathematically, it is represented as a set of layers of linearly interconnected neurons, which optimally exchange weighted information between them. Weights can be fine-tuned based on experience, making neural networks adaptive to inputs and capable of learning, and in particular conferring the ability to assign a classification based on measurable characteristics that can be used as the basic of a diagnosis. To work with a neural network, two dichotomous databases with a finite number of characteristics are required (one for training and another for testing). To achieve automatic detection of pneumonia patterns using neural networks, both training and testing must be performed. For the training step, we used a feed-forward neural network composed of 3 layers (input, hidden and output). Taking into account that increasing the number of hidden layers doesn’t significantly improve the performance of the network, this architecture was chosen. This neural network was implemented in MatLab [20] and algorithms are described in the material available at: https://osf.io/hmr3w/.

Briefly, the activation function for each neuron was implemented as a sigmoid function. Furthermore, our neural network has four neurons in the input layer; we tested several numbers of neurons in the intermediate (hidden) layer, and two neurons in the output layer. With increasing hidden neuron numbers, the prediction accuracy increases due to the greater number of training events [21]. Since no definitive rule is available to determine the number of layer or neurons to be used in a given topology, we evaluated different number of neurons (10, 50, 100, 200 and 300) in the hidden layer in a trial and error examination. The number of neurons to test in the hidden layer was selected by an empirical rule of thumb where the number of available training-data sets an upper bound for the number of processing elements, thus establishing a limit on the number of neurons in the hidden layer and preventing overfitting. This rule is described by the following equation: $N_{h}=N_{s}/(alpha*(N_{i}+N_{o}))$, where $N_{h},N_{i},N_{o}$ represent the number of neurons in the hidden, input, output layers; $N_{s}$ corresponds to the number of training samples: and alpha is an arbitrary scale factor. The alpha constant is a way of indicating how generalizable we want our model to be or how much we want to prevent overfitting. We set alpha=1 as the reference point corresponding to optimal neural network architecture [22].

The training of the neural network was performed on a set composed of 1611 vectors corresponding to 8 patients with a diagnosis of pneumonia. To avoid overfitting in our model, it was necessary to perform a wide variety of workouts; each one performed on a different training set, while randomly changing the patients that compose both our training set and our testing set for each workout. This is a standard procedure in machine learning for prevention of overfitting [23].

## Results

### Study population

Mean age for cases was 22.4 months [range: 7–50 months]; 60% female and mean age for controls was 28.6 months [range: 8–56 months]; 50% female.

Each ultrasound exam had a mean duration of approximately 10 minutes.

### Viewer classification

We analyzed 60 ultrasound frames, including 30 frames from 15 patients diagnosed with pneumonia, and another 30 frames without evidence of pneumonia from 6 patients. For training purposes we used 1611 vectors from 16 frames diagnosed with pneumonia (corresponding to 8 patients) and 17 frames showing no infection (corresponding to 3 patients). For testing purposes we used 1444 vectors from 14 frames diagnosed with pneumonia (corresponding to 7 patients) and 13 frames showing no infection (corresponding to 3 patients). All the raw data have been uploaded to OSF: https://osf.io/hmr3w/

#### Pleural line identification and skin/soft tissue removal

**Fig 5:**
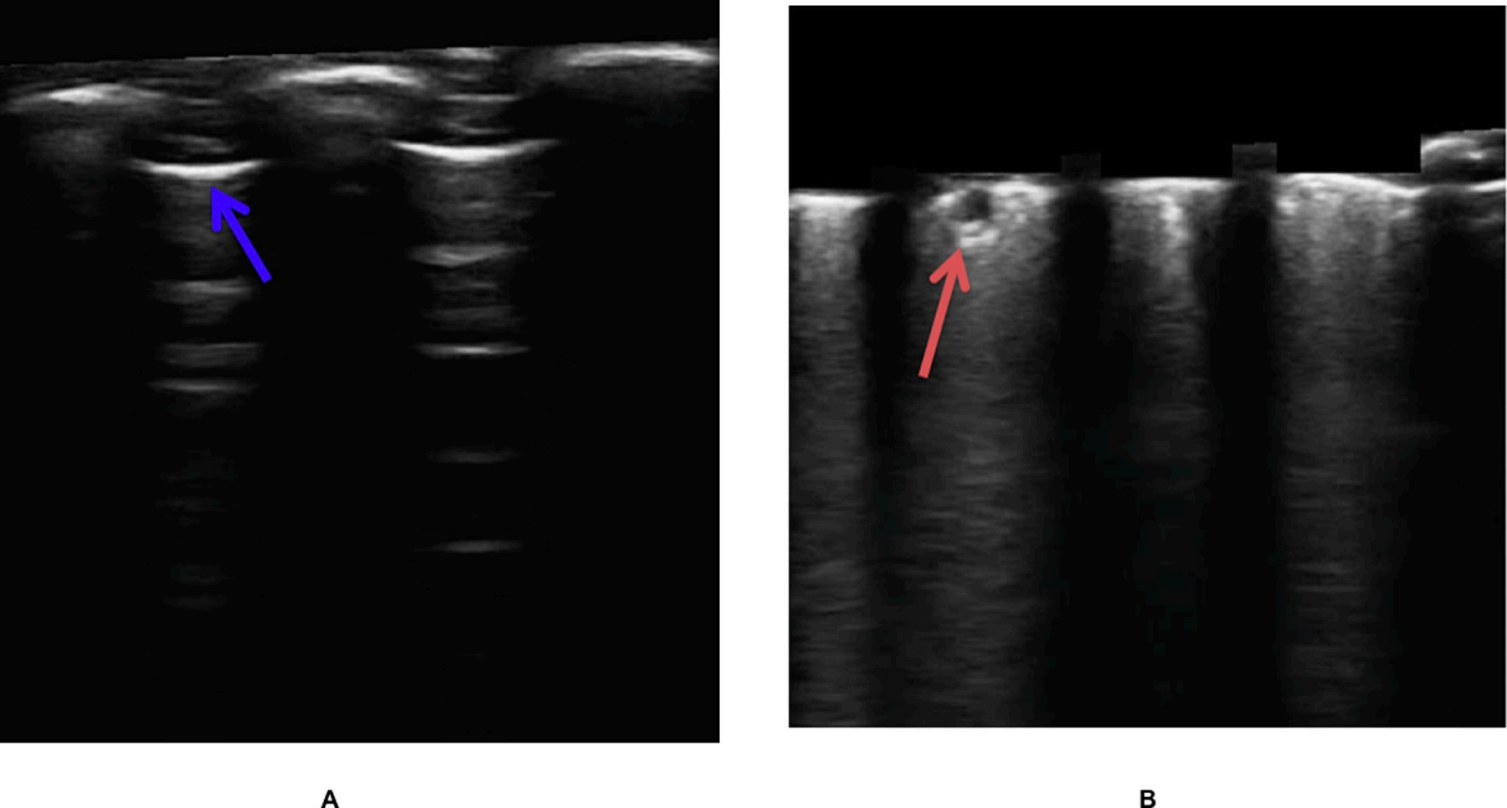
Automatic identification of the pleural line by the algorithm and removal of the skin. (A) ultrasound image of a healthy lung where the pleura is identified between two rib-bones (blue arrow), (B) ultrasound image of the lung where the soft tissues were removed and with evidence of infiltrate (red arrow).

Following the steps previously mentioned, the algorithm predicted the location of the pleural line (Fig 5A), and it was possible to remove the region coincided with (noise corresponding to the skin, muscle and other soft tissues; Fig 5B). For 59 of the 60 frames, the pleural line estimated by the algorithm was very close to the pleural line drawn by the expert analyst. In one frame the algorithm failed to accurately detect the position of the pleural line. The mean quadratic error was 11.17 pixels (min = 7.17, max = 13.67, STD = 1.57), which is about 1/40th of the total length of the frame (i.e. about 2.5% variation of the total length). In all cases the pleural line estimated by the algorithm was on top of the line drawn by the expert, therefore it is unlikely to miss evidence of pneumonia consolidates after skin and soft tissue removal.

#### Brightness profile of vectors

**Fig 6:**
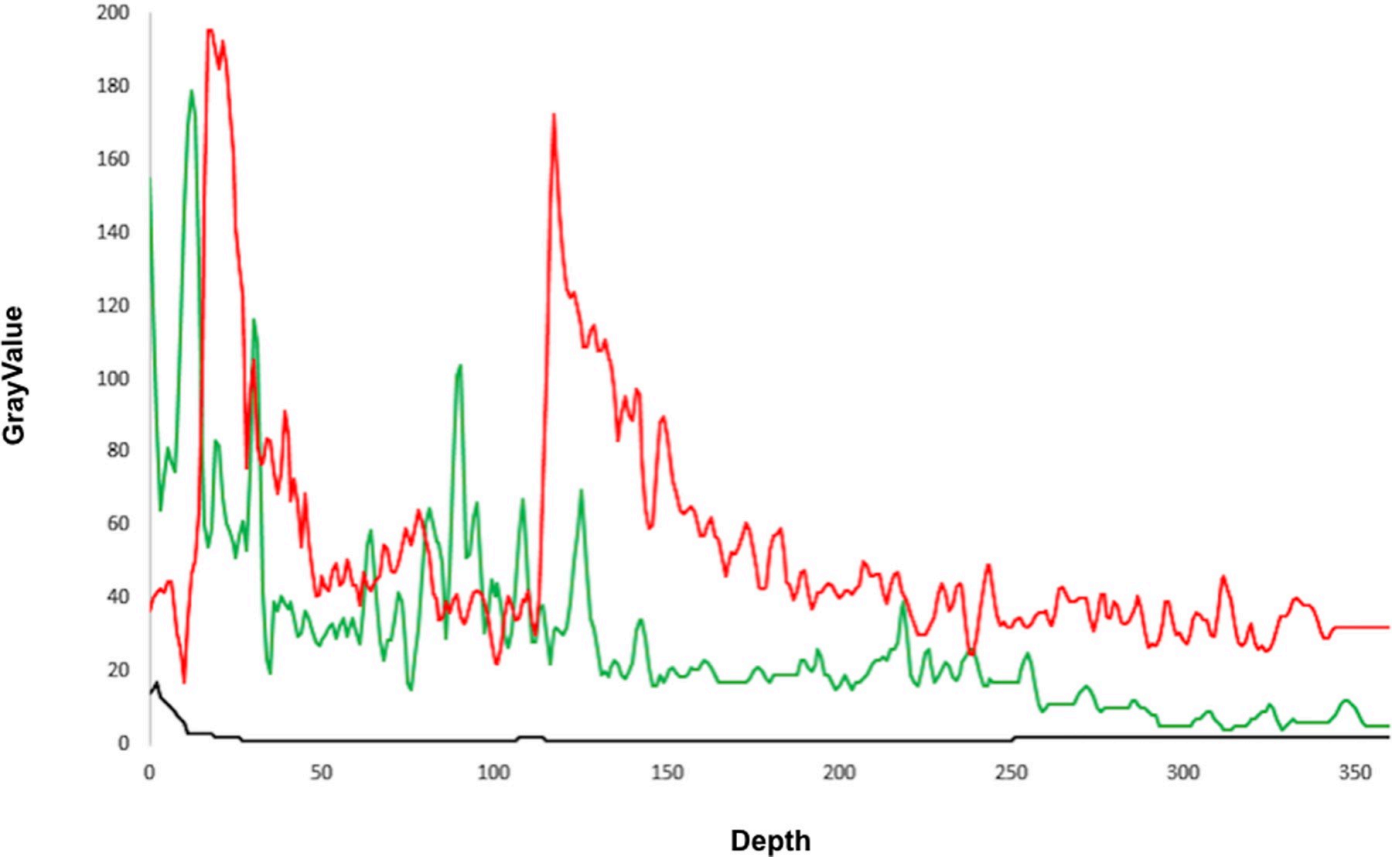
Brightness profile of an example vector with: Healthy lung (green), pneumonia (red) and rib-bone (black).

Looking at several brightness profiles, we can distinguish their characteristics associated to the three possible vector classifications: pneumonia, healthy, and bone. First, a healthy lung vector shows a smooth drop in brightness, with a very pronounced maximum followed by an exponential decay (Fig 6). These profiles may present several peaks, with values comparable to those of the pleural line, and occurring beyond the pleural–line point. From the pleural-line point, few peaks occur, and if they occur, their values are much smaller than the half of the maximum one. Second, a pneumonia vector shows a profile with mean values having an erratic behavior (Fig 6), deviating from a smooth decay. Finally, bone vectors usually show an abrupt drop in brightness from a pronounced maximum corresponding to a portion of the pleural line. In situations where the pleural line is completely removed, no drop is observed and the complete profile is almost flat (Fig 6). This corresponds to dark areas, with pixels of low brightness. The distribution of the four features corresponding to F1 (F1-25, F1-50, F1-75 and F1-100) for both the dataset corresponding to healthy tissue and to infiltrate from pneumonia, shows a bias towards higher values of brightness in the pneumonia group. This is associated with the presence of bright areas corresponding to the accumulation of water in the images with evidence of pulmonary infiltrate.

### Performance of the artificial neural network algorithm

The best classification of vectors associated with pneumonia infiltrates was performed by the neural network trained with four features (the average brightness of the sections of the vectors corresponding to the 25, 50, 75 and 100 percentiles of the length without considering the skin, starting from the first element near the pleural line towards the last one) explained above.

**Fig 7:**
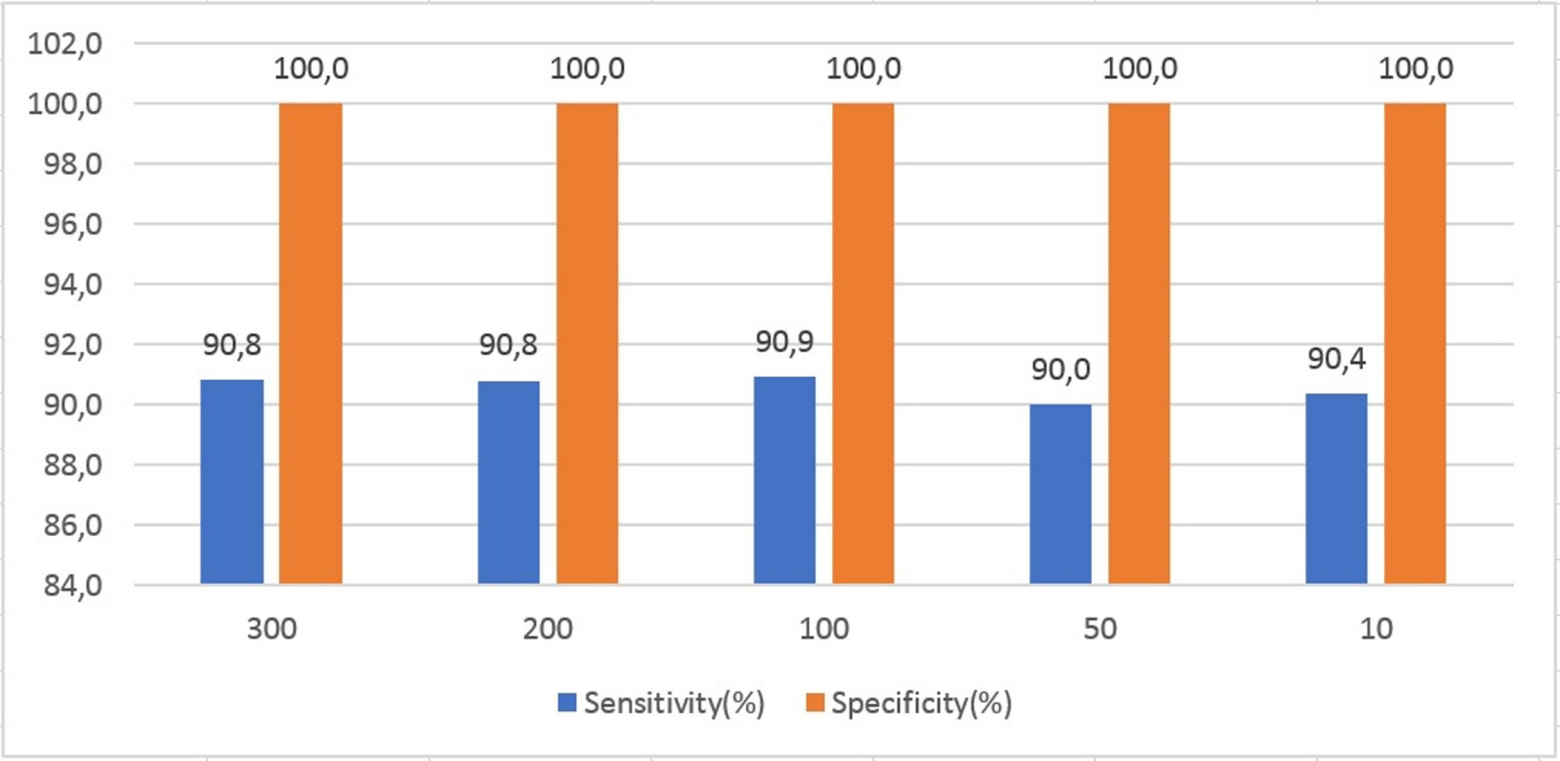
Sensitivity and Specificity in function of number of neurons in the hidden layer of the artificial neural network.

The best model corresponded to a topology of 100 neurons in the hidden layer, reaching a sensitivity of 90.9% and a specificity of 100% to correctly classify vectors (Fig 7), demonstrating pneumonia consolidation from other anatomical features (bone, normal lung), (Table 1).

**Table 1:** Performance of the artificial neural network for detection of pneumonia vectors identified by manual image analysis.

		Input	Vec	Training	Testing	Sensitivity (%)	Specificity (%)
**Features**	**F1**	4	10	1611	1444	91.52	100
	**F2**	4	10	1611	1444	90.68	100

Input: Number of predicting features/variables

Vec: Number of vector neighbors that were averaged

Training: Number of vectors used for training

Testing: Number of vectors used for testing

When analyzed by patient, patients with a diagnosis of pneumonia demonstrated an average of 50 column vectors identified as pneumonia per frame (range 31–141). No frames of patients without pneumonia demonstrated column vectors that were identified as showing pneumonia by the algorithm. Clearly, in order to estimate diagnostic parameters such as sensitivity or specificity at the level of patients, further studies in a larger sample of patients are required.

## Discussion

This study demonstrates that it is possible to train an artificial neural network to detect evidence of pneumonia infiltrates in ultrasound lung images collected from young, hospitalized children with a diagnosis of pneumonia is. Our method achieved a sensitivity of 90.9% and a specificity of 100% to detect vectors associated with pneumonia consolidates when compared to the visual recognition performed by an expert analyst.

Traditional pneumonia diagnosis relies on clinical integration of physical exam, chest X-rays and laboratory tests. However, physical exam of a tachypneic, febrile infant can be challenging, even for trained health workers; besides, chest radiographs and laboratory tests are costly and often not available in resource-limited settings. An algorithm like the one developed in this study proves that it is possible to develop a methodology to automatically detect lung infiltrates due to pneumonia in young children using ultrasound; moreover, this technology might be applied to more portable and less expensive ultrasound devices and be taken to remote, rural areas, where diagnosing pneumonia is frequently a challenge. In addition, this algorithm could also be programmed in portable low cost printed circuit boards, low-cost computers, or mobile phones, to work with portable and currently available ultrasound systems. This approach could result in an easy to carry and field-friendly system for detection of pneumonia in children, in low resource settings where experts in ultrasound images interpretation are often not present.

Although our algorithm was developed from images of a specific ultrasound device (Ultrasonix Touch) it can be adapted to other ultrasound devices, as the principle in which the algorithm is based is the same for high-tech and less sophisticated ultrasound devices.

This proof of concept study will require further development for clinical application, and our data are subject to certain limitations: all the children with pneumonia were admitted patients with lobar pneumonia, which means that severe and radiographically-evident cases were analyzed for this study; this may affect the performance of the algorithm in less severe cases, where a lower sensitivity is expected.

It is important to highlight that in order to reduce overfitting, we made sure that vectors distributed in the training/testing set, corresponded to a single patient. Therefore, vectors from a same patient were never considered being part of both the training and testing set, because neighboring vectors are likely to carry a similar information.

Further studies are needed to determine if other patterns of pulmonary disease, such as interstitial infiltrates or bronchiolitis, can be distinguished from lobar pneumonia using this approach. Furthermore, the algorithm presented here targets the vectors as the unit of analysis. In order to apply this technology to detect individuals with pneumonia (focusing on the patient rather than the image or vector as the unit of analysis), further evaluation and a larger number of cases are required.

Although this is a promising result, that opens possibility of further studies with a larger sample of children, it is important to highlight that the results presented here are valid for a single sonographer model. It is expected that results may change depending of the quality of the ultrasound images. Further studies to generalize an algorithm as the one presented here would be required to validate it for different ultrasonographers.

In conclusion, a high sensitivity and specificity were achieved for the classification of characteristic vectors associated with pneumonia infiltrates versus vectors with no evidence of pneumonia infiltrates. This is a promising tool to further develop and potentially improve the diagnosis of pneumonia, especially in rural regions where access to accurate diagnosis is poor. This would require further testing with a larger number of children involved, having each child as the unit of analysis. This approach has the possibility of reducing deaths if data is adequately processed to make automatic interpretation feasible for a larger group of patients in resource-limited areas of the world, where the lack of diagnostics for pneumonia is critical.
